# Personalized medicine: FAQs

**DOI:** 10.4103/0971-5851.71661

**Published:** 2010

**Authors:** T. Rajkumar

**Affiliations:** *Department of Molecular Oncology, Cancer Institute (Wia), Adyar, Chennai, India*

## 1. WHAT IS PERSONALIZED MEDICINE?

Currently, an individual is treated for a particular disease, based on the data obtained from thousands of other patients. A physician presumes that the new patient would also respond to the drugs which he prescribes. However, not always, do these presumptions turn out to be correct. Additionally, there could be instances wherein the treatment may produce severe side-effects, sometimes even leading to fatal consequences. Again, while dose-related side-effects are easier to predict, others may not be so.

The genomic revolution now promises to help identify the most appropriate drug for a disease for a given individual, not only ensuring activity but also avoiding those that can cause toxicity. Thus, the medical care is now tailored to an individual’s characteristics (genomic information) and can be called as Personalized Medicine.

## 2. WHAT IS THIS GENOMIC INFORMATION?

**Figure 1:**
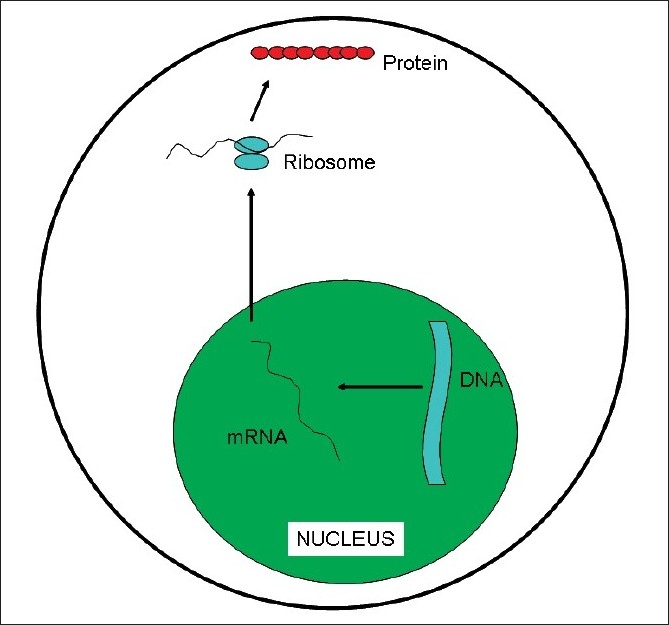
DNA to RNA to protein

Genome can be defined as the sum of all the genetic information in a given individual. We all know that the alphabets for the DNA are Adenine, Guanine, Cytosine and Thymine (A, G, C, T). It is the way these alphabets are stringed together which determines the gene sequence. When a particular gene needs to be expressed as a protein, its DNA sequence is read out as a messenger RNA (mRNA), which in turn moves from the nucleus of the cell to the cytoplasm wherein it is then translated into protein [Figure 1]. In the mRNA, Uracil replaces Thymine. The machine which does the translation of mRNA to protein is called ribosome and it does this by reading three alphabets at a time (called as triplet codon). Each codon codes for an amino acid which are the building blocks for the proteins – thus AUG codes for the amino acid Methionine, ACG codes for Threonine, AUC codes for Isoleucine, AAU for Asparagine and so forth. There are also codes for starting the translation (initiation codon, which is AUG coding for Methionine) and stopping the translation (stop codons which are UGA, UAG, UAA) of the mRNA, which ensures that the right length protein is synthesized. Currently, it is presumed that there are about 25,000 genes in the humans. However, a large number of genes have different/alternate start codons which can result in different sized protein variants (transcript variants), giving rise to additional complexity.

As humans, all of us have a similar genetic code, which is nearly 99.9% similar. The 0.1% difference is what makes us different, you being you and your friend being him. These differences are due to change in the DNA sequence, sometimes a mere change of one alphabet. This is called as Single Nucleotide Polymorphisms or SNPs. The effect of one alphabet change can be variable; some could have a profound change in the meaning/function while others may have minimal or no effect, as shown below:

e.g. Cattle – Battle; Tumour – Tumor

**Table:** 

DNA code	Amino acid
TGG	Tryphtopan
TCG	Serine
TTG	Leucine
TAG	STOP

In the example shown above, a single alphabet change in the second position of the codon leads to changes in the amino acid and worse can lead to prematurely stopping the translation of the protein which is likely to be functionally inactive. The latter is called as Nonsense mutation while the others are called as missense mutations.

## 3. WHAT ARE THE EFFECTS OF THE GENETIC MUTATIONS/POLYMORPHISMS?

There are three possible effects on the protein function:

- no effect on the protein function or
- decreased efficiency of protein function or
- increased efficiency of protein function.

**Figure 2:**
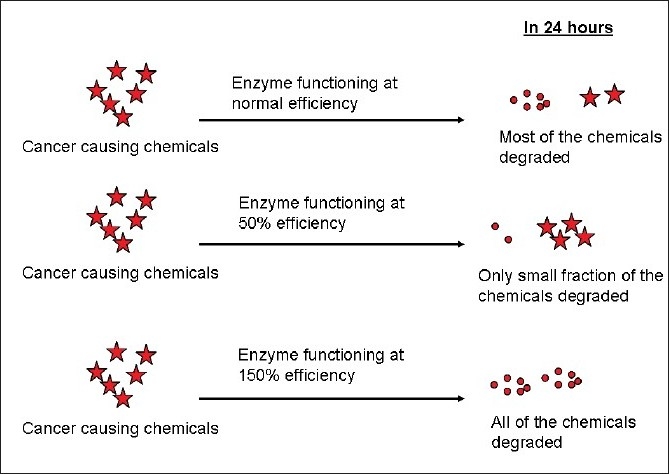
Effect of SNP on protein function

Let us take a gene which codes for a protein which is involved in the metabolism of certain cancer causing chemicals. This protein could be involved in breaking down the harmful cancer causing chemicals to harmless compounds which can then be excreted by the body [Figure 2]. If there is an SNP in the gene and if it alters the function such that this protein functions only at 50% of its inherent efficiency, then the cancer causing chemicals will be present in the body for a longer duration, leading to an increased risk for the development of cancer; on the contrary, if the protein functions at 150% efficiency, then the cancer causing chemical is rapidly broken down, resulting in a lower risk for cancer (this could explain why some smokers do not develop cancers).

## 4. HOW DOES THIS TRANSLATE TO PERSONALIZED MEDICINE?

As mentioned earlier, every one of us has SNPs in different genes and different DNA sequences. Work is progressing fast to study the associations of the SNPs with different diseases.

Additionally, we also know about certain genes which are specifically linked to specific diseases, such as *BRCA1* and *BRCA2* associated with breast and ovarian cancers. It is possible to identify disease causing mutations in these genes and offer preventive strategies in the carriers of these disease causing mutations.

While hereditary cancers account for only 5–10% of cancers, 90–95% of the cancers that are likely to be seen occur by chance (sporadic cancers). The SNPs can help here also in predicting those at risk. Unlike the high risk associated with *BRCA1* or *BRCA2*, these SNPs are low risk entities. However, it is likely that in a given individual there could be more than 50 SNPs which could alter the function of the proteins significantly. Even if they constitute a 1% risk individually, a sum of 50 genes could add upto 50% increased risk for a particular disease (e.g. breast cancer). Thus, preventive strategies will need to be applied to this large population as well.

It will be also possible to identify individuals who are likely to respond to a particular drug or who could develop undue toxicity due to a drug. For example, one of the drugs used in the treatment of blood cancers is 6-mercaptopurine. This drug is metabolized by an enzyme Thiopurine methyl transferase (TPMT) into inactive metabolites. A polymorphism in this gene results in a functionally deficient TPMT enzyme, which can lead to severe toxicity since 6-mercaptopurine is not being converted to inactive form. In such individuals, it is essential to reduce the dose of the drug.

Thus, personalized medicine can help in preventing diseases and in choosing the most appropriate drugs with best activity and least side-effects for a given individual.

## 5. HOW CAN WE IMPLEMENT PERSONALIZED MEDICINE?

The human genome was completely sequenced in 2003. The technology for DNA sequencing is improving and becoming cheaper. Currently, it would cost Rs. 4.00 to 5.00 lakhs to do a whole genome sequencing. However, in 5–10 years time, this is expected to fall to Rs.50,000 or less. Thus, every newborn can have his/her blood sample collected soon after birth and the whole genome sequencing done. This will then help in the following ways.

- a. To identify diseases which the baby is likely to be predisposed to during his/her lifetime. This will help in initiating strategies to prevent the development of the disease or reduce that risk substantially.
- To identify drugs which are likely to be toxic/cause severe side-effects, so that these drugs can be flagged in the baby’s record and avoided if there be a need later.
- To identify drugs which are more likely to be effective for diseases manifested later during life, and with minimal side-effects.
- To tailor the treatment, based on the biological characteristics of the cancer, if one were to develop it later in spite of preventive strategies. This will ensure that the most effective drugs targeted to the specific cancer are given, increasing the cure rates with minimal side-effects.

Thus, instead of casting a traditional horoscope soon after birth, it would be possible and prudent to get the Genomic Horoscope done for the baby.

## 6. WHEN WILL THIS ERA BE POSSIBLE?

With the rapidly evolving technology and more and more data being generated on SNPs and their disease associations, it should be possible to reap the benefits in 5–10 years time.

## 7. WHAT ARE THE LIMITATIONS OF THIS APPROACH?

Currently, we have the complete human genome sequence. However, we do not yet have all the information on the proteins; in particular, we do not yet know the functional effects of all the SNPs on the proteins. Additionally, we do not yet have information on all the transcript variants, and hence, our protein-related predictions could be limited.

However, with the focus now shifting from genome to proteome, in 10 years time with better technology, it should be possible to overcome these limitations.

I have not mentioned cost as a limiting factor here – a cost of Rs.50,000 to get the complete genome sequencing will be a fraction of the cost of the benefits reaped, particularly in preventing or delaying the onset of disease. This together with the ability to choose the best drug for a disease and to avoid side-effect causing drugs will ensure that the sequencing would be a boon. It would be in the interest of the Government to ensure that this is done on the same scale as the Unique Identification Number for all the subjects of the country and have the information embedded in the card itself.

## 8. ARE THERE ANY OTHER ISSUES?

As with any genetic testing, ethical issues will be raised, in particular, about the privacy. This is likely to be linked with insurance, as insurance companies may increase their premiums based on the susceptibility data. However, a clear guideline by the Government will help ensure that people are not deprived of the benefit likely to accrue in the long term.

## 9. WHERE ARE WE IN THIS ROAD MAP TO PERSONALIZED MEDICINE CURRENTLY?

We have moved a fair distance now. In oncology, more and more cancers are being treated based on the individual tumor characteristics and not merely by the generic label (e.g. breast cancer). As an example, chronic myeloid leukemia, a blood cancer which has a specific gene abnormality (translocation of *abl* gene from chromosome 9 to chromosome 22, resulting in a novel gene, with the resultant novel protein) is now treated with a drug which targets this abnormality (Imatinib). This is a paradigm shift, since earlier chemotherapies destroyed not only the cancer cells but also the normal cells. Newer drugs which target cancer specific abnormalities are currently being developed.

Better biological characterization of breast cancer has helped to identify subtypes which are likely to respond differently/poorly to the current drugs. This can help choose better drugs for these difficult subtypes. Additionally, drugs which can help prevent development of breast cancer in high risk *BRCA1* or *BRCA2* mutation carriers are being evaluated and these are likely to have fewer side effects compared to Tamoxifen which can be used for this purpose.

The use of a subject’s/patient’s genomic data for predicting toxicity to a drug (as explained earlier) is also in vogue now.

## 10. IS THIS ARTICLE A MERE SCIENCE FICTION?

As pointed out above, we are already moving into Personalized Medicine era. The technologies being developed are more and more powerful and likely to bring the cost down considerably. Hence, this is not a pipe dream but a road map to the future.

